# Spiro[pyrrolidine-3,3′-oxindoles] and Their Indoline Analogues as New 5-HT6 Receptor Chemotypes

**DOI:** 10.3390/molecules22122221

**Published:** 2017-12-14

**Authors:** Ádám A. Kelemen, Grzegorz Satala, Andrzej J. Bojarski, György M. Keserű

**Affiliations:** 1 Medicinal Chemistry Research Group, Research Centre for Natural Sciences, Hungarian Academy of Sciences, Magyar tudósok körútja 2, H1117 Budapest, Hungary; kelemen.adam@ttk.mta.hu; 2 Department of Medicinal Chemistry, Institute of Pharmacology, Polish Academy of Sciences, 12 Smętna Street, 31-343 Krakow, Poland; grzegorz.satala@gmail.com (G.S.); andrzej.bojarski@gmail.com (A.J.B.)

**Keywords:** oxindole, indoline, coerulescine, 5-HT_6_R, G-protein coupled receptor

## Abstract

Synthetic derivatives of spiro[pyrrolidinyl-3,3′-oxindole] alkaloids (coerulescine analogues) were investigated as new ligands for aminergic G-protein coupled receptors (GPCRs). The chemical starting point 2′-phenylspiro[indoline-3,3′-pyrrolidin]-2-one scaffold was identified by virtual fragment screening utilizing ligand- and structure based methods. As a part of the hit-to-lead optimization a structure-activity relationship analysis was performed to explore the differently substituted 2′-phenyl-derivatives, introducing the phenylsulphonyl pharmacophore and examining the corresponding reduced spiro[pyrrolidine-3,3′-indoline] scaffold. The optimization process led to ligands with submicromolar affinities towards the 5-HT_6_ receptor that might serve as viable leads for further optimization.

## 1. Introduction

**Figure 1:**
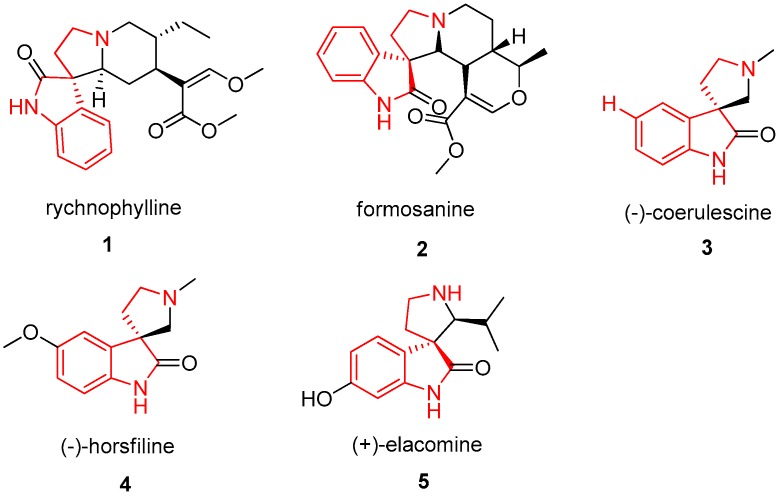
Representative natural spiropyrrolidinyl-oxindole alkaloids. The tryptamine scaffold is shown as red skeleton.

The recent isolation of naturally occurring and biologically active spiropyrrolidinyl-oxindole alkaloids initiated a significant research on synthetic derivatives [1], particularly compounds with the spiro[indoline-3,3′-pyrrolidine]-2-one ring system. Spiropyrrolidinyl-oxindole alkaloids were isolated from different species including *Gelsemium sempervirens*, *Aspidosperma*, *Mitragyna*, *Ourouparia*, *Rauwolfia*, *Vinca* species [2]. The most prominent examples are rynchophylline (**1**), formosanine (**2**), coerulescine (**3**), horsfiline (**4**) and elacomine (**5**) that show diverse biological activity (Figure 1). Although these compounds have a common tryptamine derived motif, a ChEMBL-analysis [3] revealed that no representatives have been ever tested against serotonergic targets. Developing ligand-(FrAGs) [4] and structure-based (FrACS) [5] methods for the identification of aminergic receptor ligands we identified **6** as a micromolar 5-hydroxytryptamine receptor 6 (5-HT_6_R) ligand.

**Figure 2:**
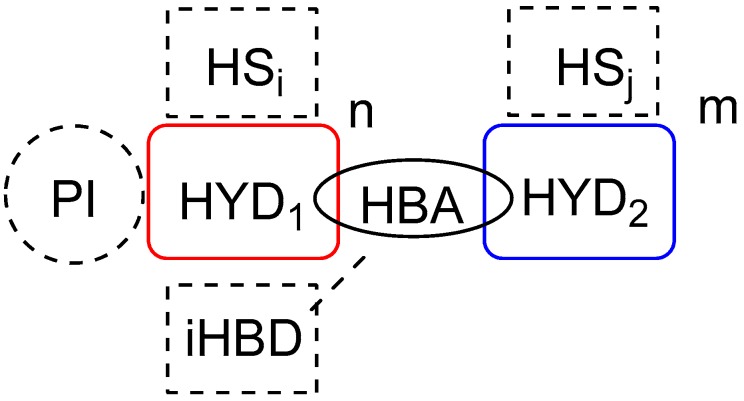
General pharmacophore model of 5-HT_6_R antagonists. PI: positive ionisable portion; HYD_1,2_: hydrophobic sites; iHBD: intramolecular hydrogen bond donor site; HBA: hydrogen bond acceptor feature; HS_i,j_: substituents on the hydrophobic sites.

The 5-HT_6_R is a member of the Class A G-protein coupled receptors (aminergic family) considered to be a current and promising drug target for the treatment of several central nervous system related indications, such as: cognitive, learning and memory deficits related to Alzheimer’s disease [6], Parkinson’s disease [7] and schizophrenia [8]. Chemical similarity to the endogenous agonist serotonin explains the most frequent heteroaromatic ring systems routinely used in 5-HT_6_R ligands that include indoles, indolines, indazoles, pyrrolo[2,3-*b*]pyridines, pyrazolo[1,5-*a*]pyridines, [1,2,3]-triazolo[1,5-*a*]pyrimidines and further 5 + 6 condensed *N*-heterocycles. Pharmacophore based approaches on 5-HT_6_R antagonists are typically focused to the canonical pharmacophore model [9] (Figure 2), which is defined as having two hydrophobic rings/ring systems connected by a hydrogen bond acceptor (e.g., sulfonyl, sulfonamide linker). Optionally, a positively ionizable residue is included in one of the hydrophobic sites of the ligands, typically offering an interacting moiety with the D^3.32^ aspartate residue of aminergic GPCR’s as a key factor of aminergic 7TM-receptor activation [10]. A further pharmacophore feature might contain an additional intramolecular hydrogen bond donor moiety further stabilizing the binding conformation of the ligands [11] (see Figure 2). Selectivity among other aminergic GPCR’s was shown [12] to be accessible through omitting the positively ionizable group in the 5-HT_6_R antagonists. Bis(hetero)arylsulphonyl- and sulfonamide substituents also contribute to 5-HT_6_R affinity and selectivity.

The tryptamine derived scaffold of **6** and its alignment with the published 5-HT_6_R pharmacophore [9] prompted us to explore the structure-activity relationship around the spiropyrrolidinyl-oxindole core. Here we report the results of our hit-to-lead program, which led to an identification of promising 5-HT_6_R ligands of this chemotype.

## 2. Results and Discussion

### 2.1. Identification and Early Structure-Activity Data on Spiro[pyrrolidine-3,3′-oxindole] Derivatives

Fragment libraries containing a couple hundreds or even thousands of small polar molecules are routinely used for hit identification at the early stage of drug discovery. Fragment screening provides diverse chemotypes and significant operational freedom for the further optimization of promising hits. Inspired by these advantages in a recent study we developed a strategy for aminergic focused fragment libraries using a sequential filtering methodology applying ligand- and structure-based scoring functions [4,5]. The prospective validation was performed on our in-house library of 1183 fragments. A physicochemical-property based scoring, followed by docking the fragments into an ensemble of carefully selected aminergic GPCR X-ray structures (PDB ID: 3PBL, 3RZE, 4IB4, 4IAQ, 3UON, 4MQT, 4LDE, 2RH1, 3NY9). This resulted in a set of 36 top ranked hit molecules which were measured on an aminergic target not being included in the original docking ensemble, namely 5-HT_6_R. We demonstrate the usefulness of the method for comprehensive aminergic focused screenings. Out of the four hits with low micromolar inhibitory results, the structurally novel 2′-(3-fluorophenyl)spiro[indoline-3,3′-pyrrolidin]-2-one (**6**) possessing the spiro[pyrrolidine-3,3′-oxindole] scaffold (Table 1), was selected for further optimization.

As the next step of exploring the structure-activity relationship substructure search in the MCULE purchasable database [13] of 5 million compounds resulted in further 887 spiro[pyrrolidine-3,3′-oxindole] derivatives. The molecules were prepared by Schrödinger’s LigPrep [14] creating possible conformers, tautomers and protonation states by default settings. The ligands were then projected to single precision molecular docking analysis on a nine membered ensemble of molecular dynamics frames of an 5-HT_6_R homology model [15] (built using 5-HT_2B_R X-ray crystal structure [16] in complex with ergotamine as template (PDB ID: 4IB4)). The docking poses were filtered in a post-processing step keeping only binding modes forming hydrogen bonds towards Asp106^3.32^, Asn288^6.55^ and optionally Ser193^5.43^, occupying a primary hydrophobic cleft defined by Trp281^6.48^, Phe284^6.51^ and Phe285^6.52^. A secondary hydrophobic subpocket was defined by the pose filtering criteria as following: Val107^3.33^, Ala157^4.56^, Leu160^4.59^, Pro161^4.60^, Leu164^4.63^. The satisfactory poses of the molecules were scored to have the best ranking in possibly all of the 9 frames using consensus ranking [5]. Altogether ten compounds (**7**–**16**) were purchased and tested in our serotonergic panel (Table 2).

**Figure 3:**
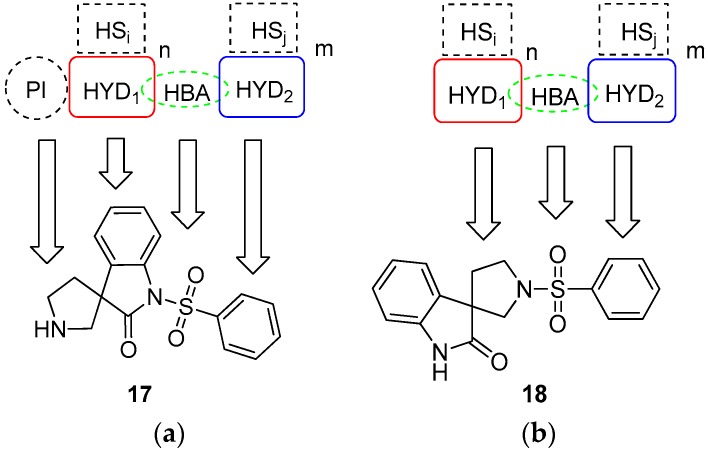
(**a**) 1-(phenylsulfonyl)spiro[indoline-3,3′-pyrrolidin]-2-one (**17**) superimposed onto the classical pharmacophore; (**b**) 1′-(phenylsulfonyl)spiro[indoline-3,3′-pyrrolidin]-2-one (**18**) fitting to the pharmacophore pattern lacking a positive ionisable moiety.

In spite of the structural diversity, all virtual screening hits were lacking substitution at the oxindole scaffold. However, compounds **7**, **9** and **10** having oxygen-containing substituents in the 3,4-positions of the 2′-phenyl ring showed somewhat improved affinity compared to the initial compound **6**. The moderate improvement in affinity and the lack of selectivity amongst the closely related serotonergic targets have driven us to interpret the results in the context of the known 5-HT_6_R pharmacophore patterns [17] (Figure 3). This analysis suggests that introducing a phenylsulfonyl moiety either to the 1-nitrogen at the oxindole ring or to the 1′-nitrogen at the pyrrolidine ring would be beneficial for both the affinity and selectivity of this chemotype.

### 2.2. Hit-to-Lead Optimization of Spiro[pyrrolidine-3,3′-oxindoles]

**Scheme 1:**
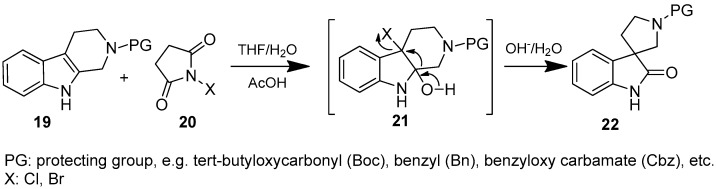
Mechanism of the succinimide assisted oxidative spiro-rearrangement of tryptolines.

Detailed elaboration of the spiro[pyrrolidine-3,3′-oxindole] scaffold required a viable synthesis strategy for the preparation of designed analogues. The first, conventional approach is based on an intramolecular Mannich-reaction used in case of several alkaloids including (±)-horsfiline (**4**) [18] and Spirotryprostatin B [19]. An alternative approach is the oxidative reaction of tryptolines induced by *tert*-butyl hypochlorite, *N*-bromosuccinimide, *N*-chlorosuccinimide, sodium tungstate, lead tetraacetate, or osmium tetroxide [20,21]. The reaction is completed by the subsequent elimination of water that finally results in the reorganization of the ring system to spiro[pyrrolidine-3,3′-oxindoles] (Scheme 1).

Further, sophisticated approaches such as [1,3]-dipolar cycloaddition reactions of azomethine ylides [22], radical cyclization by AIBN [23], intramolecular Heck reaction [24] and asymmetric nitroolefination of oxindoles [25] are also available, however, used less frequently.

For the effective exploration of the structure-activity relationship we need a feasible and universal approach with acceptable functional group tolerance and high variability around the spiro-oxindole core. Considering the requirements of the early stage optimization program we aimed a synthesis strategy that

- uses readily accessible, simple starting materials
- applies well-documented, readily available reagents
- has key intermediates to access a wide variety of derivatives
- is not necessarily stereoselective at this stage of the optimization

**Scheme 2:**
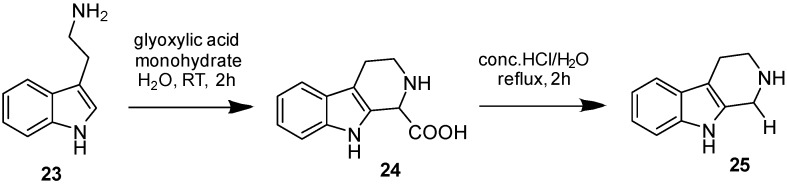
Synthesis of tryptoline by Pictet-Spengler condensation.

The oxidative spiro-rearrangement reactions offer a wide variety of oxidative reagents [20,21,26,27,28,29,30,31,32,33] and the corresponding starting materials are readily accessible through Pictet-Spengler reaction of tryptamines [34] (Scheme 2).

**Scheme 3:**
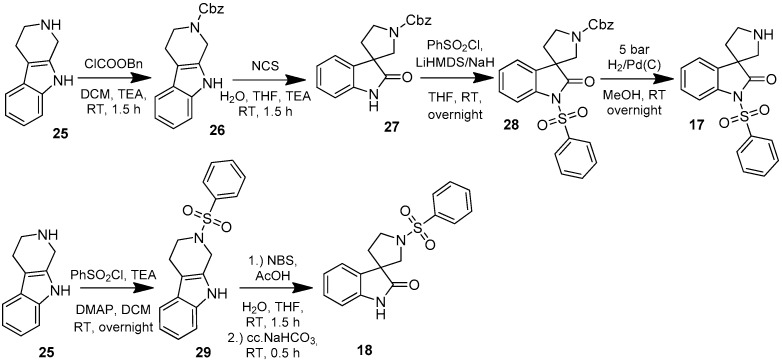
Synthesis of 1- and 1′-phenylsulfonyl derivatives.

Following this synthesis strategy, tryptamine (**23**) was used as a starting material for the synthesis of the common intermediate tryptoline (**25**). The Pictet-Spengler condensation reaction was performed by glyoxylic acid-monohydrate in aqueous medium [35]. The crude acid intermediate **24** was decarboxylated in refluxing concentrated hydrochloric acid affording the tetrahydro-β-carboline. The first synthetic route depicted in Scheme 3 starts with the *N*-acylation of the tryptoline (**25**). The application of Cbz (carboxybenzyl) [36] protecting group was necessary for two reasons: 1. achieve the sulfonylation at the oxindol-nitrogen, 2. the spiro-rearrangement reaction is not occurring in case of unsubstituted pyrido-nitrogen. *N*-chlorosuccinimide [37] was used in the first experiments as halogenating reagent, providing the Cbz-protected spiro[pyrrolidine-3,3′-oxindole]. The phenylsulfonylation was performed either by using lithium-hexamethyl disilazane [38] and sodium hydride [39] as deprotonating agents and acid scavengers. Finally, the deprotection of the Cbz-group by hydrogenation [40] resulted in the desired 1-(phenylsulfonyl)spiro[indoline-3,3′-pyrrolidin]-2-one (**17**) compound. Following an alternative way, the more basic pyrido-nitrogen of tryptoline **29** might be first sulfonylated [41] followed by the *N*-bromosuccinimide assisted spiro-cyclization to **18** [42].

Compounds **17** and **18** showed improved selectivity towards the 5-HT_6_R (Table 3) as compared to the non-sulfonylated 2′-phenyl derivatives, investigated in the first batch (compounds **15**–**24**), however, no improvement in the binding affinity was detected.

Facilitating the potency optimization, we followed the classical 5-HT_6_R pharmacophore pattern and tried to remove the polar interacting hydrogen bond acceptor feature in the oxindole ring of **17**. For better understanding of the indole, indoline and oxindole-related chemical space of known 5-HT_6_R ligands we collected all compounds, with at least 0.1 μM activity towards h5-HT_6_R from the ChEMBL database.

**Figure 4:**
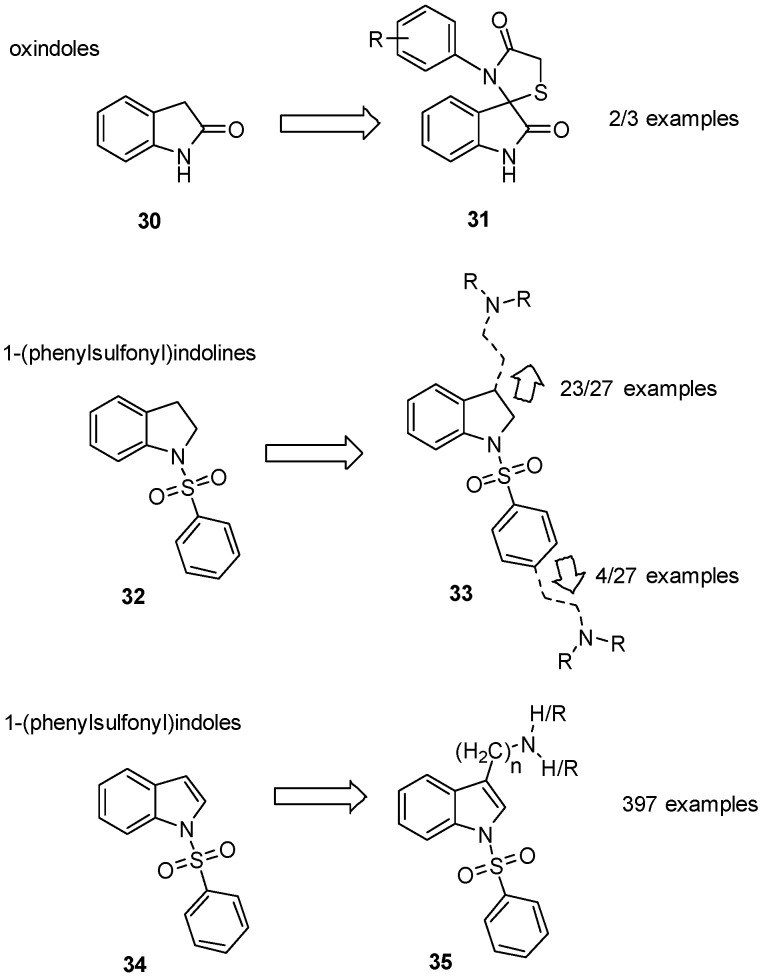
Substructure analysis of ChEMBL 5-HT_6_R active compounds.

Substructure filtering by the query of **30** (Figure 4) revealed that altogether 3 examples were found for oxindoles, as 5-HT_6_R ligands. All hits belong to the 3′-phenylspiro[indoline-3,2′-thiazolidine]-2,4′-dione scaffold **31** that was identified by Hostetler et al. [43]. Searching for the 1-(phenylsulfonyl) indoline scaffold **32**, 27 actives were found with the structure **33**. Out of the 27 active molecules 4 possess a positive ionizable moiety built upon the phenyl side of the sulfonyl and all of the remaining examples are equipped by the classical tryptamine-type amine feature. Searching by the 1-(phenylsulfonyl)indole **34** query resulted in 397 examples with the structure **35**. The structures were analyzed based on the chain-length distance between the 3-carbon of the indole ring and the positive ionizable basic nitrogen atom, if any. We found 20 examples representing indoles with attached basic nitrogen (all tertiary amines). Only five compounds were identified with a single methylene linker out of which 2 were primary amines and 3 were tertiary amines. The largest part of the with structure **34** contains ethylene linker (201 examples out of 397) of which 123 were tertiary amines, 51 were secondary amines and 19 were primary amines. Much less compounds have 3 atom long linkage, 54 examples were identified, with 21 tertiary amines and 33 secondary amines and no primary amines. No ligands were found with basic nitrogen’s being further than 3 atoms from the indole core.

**Scheme 4:**
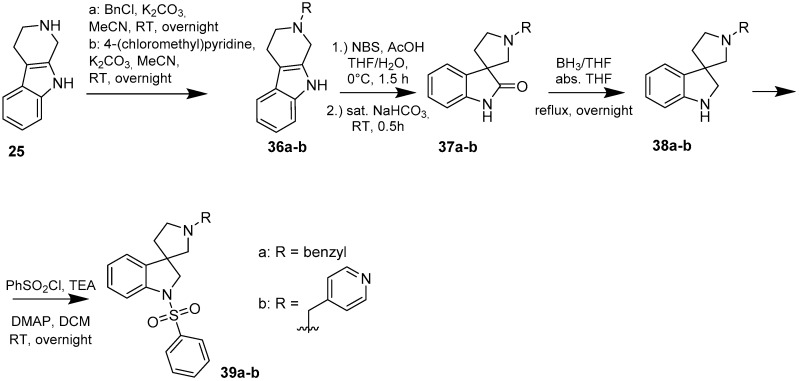
Synthesis of N′-substituted and *N*-phenylsulfonylated indolines.

To conclude, we have aimed to synthesize different 1-(phenylsulfonyl)indolines (scaffold **32**), being substituted in the 1′-pyrrolidine nitrogen with either basic-nitrogen containing, or lipophilic groups, presented in Scheme 4—compounds **39a**–**b**. Based on these considerations, first we benzylated [44] the tryptoline (**25**) to afford **36a**, further converting it to the corresponding spiro-derivative [45] **37a**. The reduction of the oxindole oxo-group was performed by applying borane-tetrahydrofuran complex in refluxing absolutized tetrahydrofuran [46]. It was an important observation, that the reduction step has to precede the sulfonylation, to avoid the formation of side products when treating the sulfonamide with the boronic reagent. The phenyl-sulfonylated product **39a** was afforded either by applying LiHMDS [38] or trietylamine/dimethylaminopyridine [47] reagents. The pyridine-4-ylmethyl derivative (**39b**) was synthesized based on the same route, however, as a first step, the tryptoline (**25**) was treated with 4-chloromethylpyridine [48,49].

**Scheme 5:**
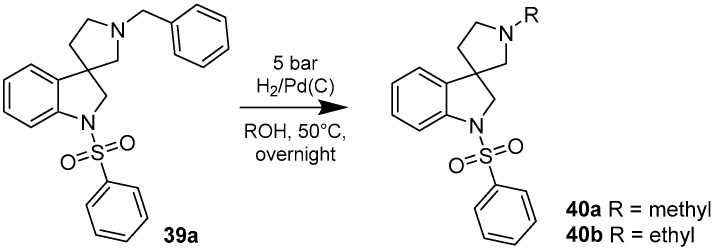
Synthesis of *N*′-methyl and ethyl derivatives.

We also intended to examine the effect of only transforming our initial test compound **17** to the corresponding reduced indoline, however the debenzylation step of **39a** afforded unexpected products (Scheme 5, **40a** and **40b**). In case, when methanol was applied as solvent for the debenzylation by catalytic hydrogenation, a methylated product **40a** has formed and respectively, the use of ethanol afforded an ethyl-alkylated derivative **40b**.

However, both the alkylated derivatives showed some improvement of binding affinity to reach the low micromolar range, the compound possessing the biggest lipophilic, bulky group **43a** has produced the best, submicromolar binding affinity, with both selectivity against the other closely related serotonergic targets (Table 4).

**Scheme 6:**
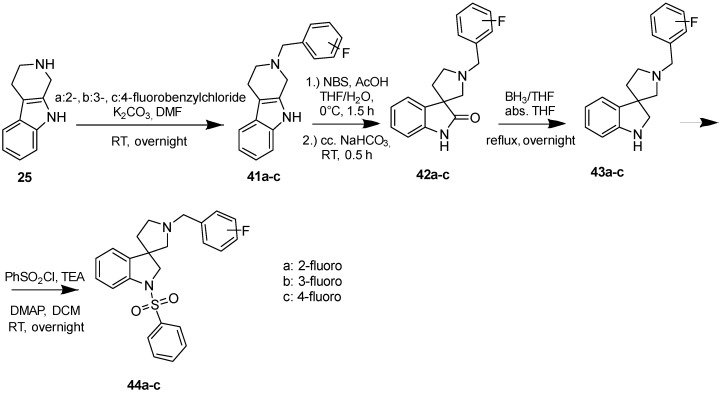
Fluoro-scan of the *N*′-benzyl direction.

The submicromolar affinity of **39a** prompted us investigating the substituent vectors at both the benzylic and the phenylsulfonyl rings by walking fluorine substituents around. This methodology, often referred as fluoro-scan has been used effectively for the identification of sites tolerant to functionalization. In particular, fluoro-scan explores the impact of enhanced lipophilicity, H-bonding and/or filling a small pocket [50]. In case of the *N*′-benzylic substitution [13] pattern, 2-, 3- and 4-fluorobenzylchlorides were used as alkylating agents in order to synthesize the corresponding fluorinated indolines (Scheme 6, **44a**–**c**).

The substitution of benzylic ring with fluorine caused a minor decrease in affinity, thus has shown, that growing towards this direction is not beneficial (Table 5). On the other hand, however, some improvement in the selectivity was observed.

**Scheme 7:**
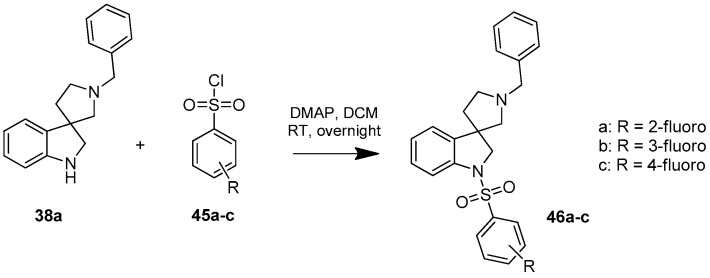
Fluoro-scan of the *N*-phenylsulfonyl direction.

The corresponding fluorophenylsulfonyl derivatives **46a**–**c** were synthesized from compound **38a** intermediate, previously synthesized (Scheme 7).

The alternative growing direction towards the phenylsulfonyl ring (compounds **46a**–**c**) showed improvement in the affinities (see Table 6), compared to the results of **39a**, also retaining the good selectivity against the 5-HT_7_ subtype. These data suggest, that position 2 of the phenylsulfonyl group of **46a** is beneficial and therefore it is worth to explore this vector during the forthcoming lead optimization program.

### 2.3. Binding Mode Analysis of the Optimized 5-HT_6_R Ligand

**Figure 5:**
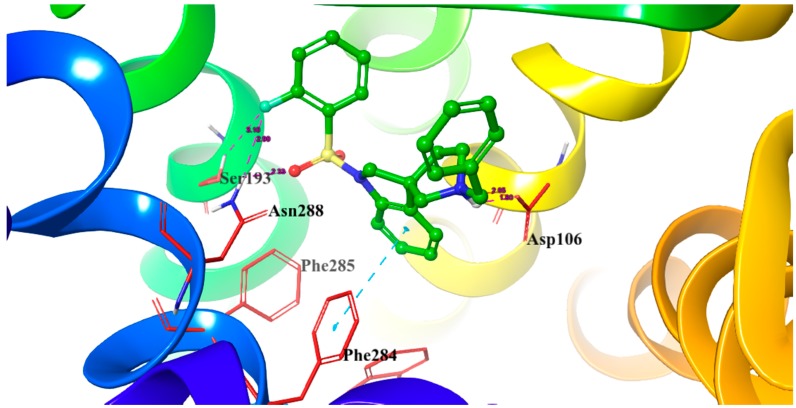
Binding mode of compound **46a** in the orthosteric pocket of 5-HT_6_R.

We have performed a docking analysis of the 1′-benzyl-1-((2-fluorophenyl)sulfonyl)-spiro[indoline-3,3′-pyrrolidine] (**46a**) 5-HT_6_R antagonist using the receptor model reported earlier [15]. Docking of **46a** by Schrödinger—Glide either with, or without applying any constraints (requiring hydrogen bonding with both Asn288^6.55^ and/or Ser193^5.43^) resulted in consistent docking poses inside the orthosteric binding pocket (Figure 5).

The indoline core occupies the primary hydrophobic cavity of the binding pocket (formed by Trp281^6.48^, Phe284^6.51^, Phe285^6.52^), while the protonated quaternary pyrrolidine-nitrogen is oriented towards the conserved Asp106^3.32^, forming a well-established hydrogen bond. The phenyl-sulfonyl moiety of **46a** is sitting at a secondary hydrophobic cleft defined by Val107^3.33^, Ala157^4.56^, Leu160^4.59^, Pro160^4.60^ and Leu164^4.63^, positioning the sulfonyl-linker as hydrogen-bond acceptor against Ser193^5.43^ and Asn288^6.55^. Interestingly, the fluorine atom as hydrogen bond acceptor is offering a possible polar interaction with the Asn288^6.55^ residue, underlining the preference of the ortho-substitution at the phenyl-sulfonyl ring.

## 3. Conclusions

**Scheme 8:**
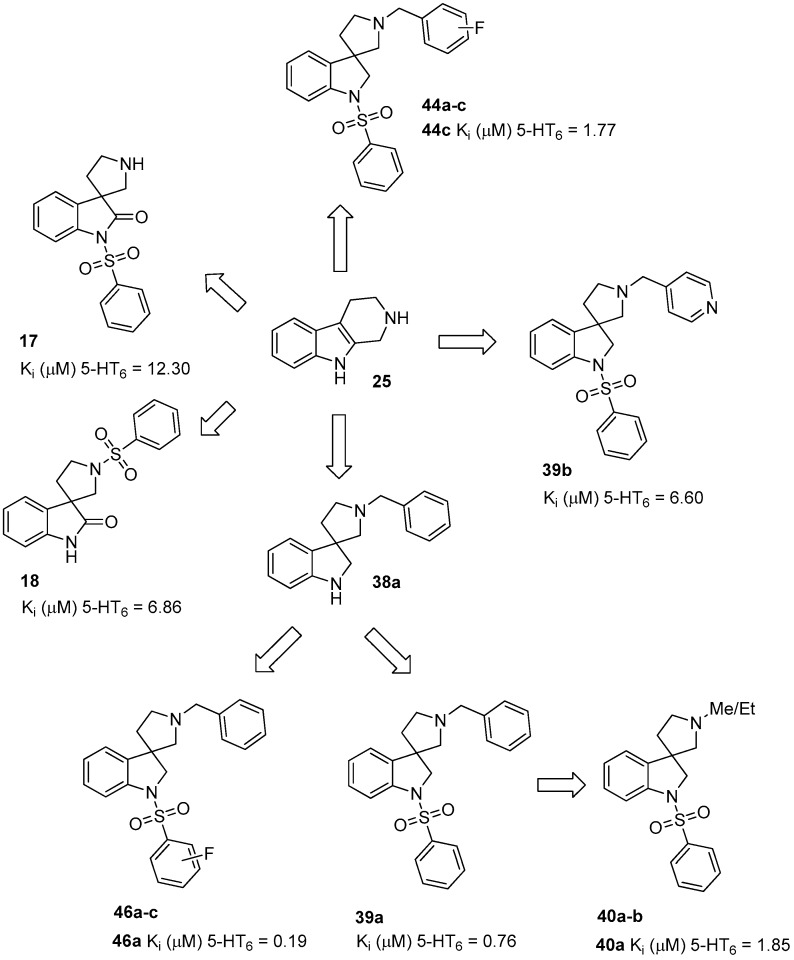
Spiro[pyrrolidine-3,3′-oxindoles] and spiro[pyrrolidine-3,3′-indolines] as 5HT6 ligands.

The structure-activity relationship around the original spiropyrrolidinyl-oxindole hit (**6**) was first investigated using the “SAR-by-catalog” approach that resulted in some moderate improvement in affinity and a low level of selectivity. Therefore, we decided to explore the novel 5-HT_6_R chemotype by a more conventional medicinal chemistry strategy. A synthetic tree (summarized in Scheme 8) of the synthesized oxindoles and indoles was elaborated starting from the core tryptoline intermediate (**25**).

The removal of the bulky lipophilic site at the 2′-position of the pyrrolidine ring and insertion of the classical phenylsulfonyl part resulted in notable selectivity across the serotoninergic GPCR panel of 4 receptors. The next optimization step was analyzing the chemistry of known indole, indoline and oxindole-based 5-HT_6_R inhibitors in ChEMBL. We have synthesized different 1-(phenylsulfonyl) indolines, being substituted in the 1′-pyrrolidine nitrogen with either basic-nitrogen containing, or lipophilic groups. As a result, 1′-benzyl-1-(phenylsulfonyl) spiro[indoline-3,3′-pyrrolidine] (**39a**) and its corresponding 2-, 3-substituted analogues **46a**–**b** were identified as 5-HT_6_R starting points. This opened a new chemical space for the under-represented spiro[pyrrolidine-3,3′-indoline] chemotype.

## 4. Materials and Methods 

All chemical reagents used were purchased from commercial chemical suppliers. The NMR experiments were performed at 500 MHz (^1^H) on a Varian VNMR SYSTEM spectrometer. Chemical shifts are referenced to the residual solvent signals, 2.50 ppm for ^1^H in DMSO-*d*_6_ and 7.28 ppm for ^1^H in CDCl_3_. The MS measurements were performed on Shimadzu LCMS2020 LC/MS system. Flash chromatography was performed using Teledyne ISCO CombiFlash Lumen+ Rf. Purifications by preparative-HPLC were performed with Hanbon NS4205 Binary high pressure semi-preparative HPLC. Thin-layer chromatography was performed on TLC Silica Gel 60 F254. High resolution mass spectrometric measurements were performed using a Q-TOF Premier mass spectrometer (Waters Corporation, Milford, MA, USA) in positive electrospray ionization mode. Compounds **7**–**16** were purchased from Mcule Inc.

### 4.1. General Procedures for the Synthesis of Spiro[pyrrolidine-3,3′-oxindole] and Spiro[pyrrolidine-3,3′-indoline] Derivatives

#### 4.1.1. Procedures to Afford 1-(Phenylsulfonyl)spiro[indoline-3,3′-pyrrolidin]-2-one (**17**)

##### Synthesis of Benzyl 2-Oxospiro[indoline-3,3′-pyrrolidine]-1′-carboxylate (**27**)

To the solution of benzyl 3,4-dihydro-1*H*-pyrido[3,4-*b*]indole-2(9*H*)-carboxylate (**26**) (918 mg, 3 mmol) in 10 mL tetrahydrofuran was added 10 mL distilled water and triethylamine (0.436 mL, 3.15 mmol, 1.05 equiv.) at room temperature. Afterwards *N*-chlorosuccinimide (431 mg, 3.24 mmol, 1.08 equiv.) was added at 0 °C in portions. The reaction mixture was stirred for 1.5 h at room temperature. The reaction mixture was diluted with 10 mL brine and was extracted using 2 × 10 mL ethyl acetate. The combined organic phases were dried over sodium-sulfate, filtered and evaporated under reduced pressure. The crude material was purified using flash chromatography (gradient: dichloromethane: methanol 0% to 5% methanol) to give **27** as a yellow solid. Yield: 399 mg (41%). ^1^H-NMR (500 MHz, DMSO-*d*_6_) δ ppm 7.42–7.28 (m, 8H, ArH-benzyl, ArH-4, ArH-5, ArH-6), 7.14 (d, *J* = 7.8 Hz, 1H, ArH-7), 5.11 (s, 2H, benzyl CH_2_), 3.70–3.53 (m, 4H, pyrrolidine H-2′, pyrrolidine H-5′), 2.19–2.05 (m, 2H, H-4′). ^13^C-NMR (125 MHz, DMSO-*d*_6_) δ ppm 176.9 (C-2), 154.8 (C=O), 142.8 (C-3a), 141.5 (C-7a), 135.9 (benzyl C-1), 128.5 (benzyl C-3, benzyl C-5), 127.9 (benzyl C-4, benzyl C-2, benzyl C-6), 123.8 (C-4), 122.1 (C-5), 109.3 (C-7), 67.9 (benzyl CH_2_), 57.7 (C-3), 45.1 (pyrrolidine C-2′), 43.3 (pyrrolidine C-5′), 34.2 (pyrrolidine C-4′). MS (DUIS+) *m*/*z* 323 [M + H]^+^.

##### Synthesis of Benzyl 2-oxo-1-(phenylsulfonyl)spiro[indoline-3,3′-pyrrolidine]-1′-carboxylate (**28**)

(1) Procedure Using Sodium-Hydride

To the solution of benzyl 2-oxospiro[indoline-3,3′-pyrrolidine]-1′-carboxylate (**27**) (175 mg, 0.542 mmol) in 5 mL anhydrous tetrahydrofuran was added sodium-hydride (60%, 28 mg, 0.705 mmol; 1.3 equiv.) at 0 °C. After stirring the reaction mixture at room temperature for 0.5 h, benzenesulfonyl chloride (0.076 mL, 0.596 mmol, 1.1 equiv.) was added dropwise at 0 °C. The reaction mixture was stirred overnight at room temperature, then it was quenched by 10 mL brine and was extracted using ethyl acetate (2 × 10 mL). The collected organic phases were dried using sodium sulfate, filtered and evaporated under reduced pressure. The crude product was purified using flash chromatography (gradient: hexane: ethyl acetate 0% to 20%) to afford **28** as a colorless solid. Yield: 94 mg (38%).

(2) Procedure Using Lithium-Hexamethyl-Disilazane

To the solution of benzyl 2-oxospiro[indoline-3,3′-pyrrolidine]-1′-carboxylate (**27**) (161 mg, 0.5 mmol) and benzenesulfonyl chloride (0.076 mL, 0.6 mmol, 1.2 equiv.) in 5 mL anhydrous tetrahydrofuran was added lithiumhexamethyl-disilazane (1M in THF, 0.85 mL, 0.85 mmol, 1.7 equiv.) at 0 °C and the reaction mixture was stirred at this temperature for 0.5 h. After quenching the reaction by 10 mL brine, the reaction mixture was extracted using ethyl acetate (2 × 10 mL) and the collected organic phases were dried over sodium sulfate, filtered and evaporated under reduced pressure. The crude product was purified using flash chromatography (gradient: hexane: ethyl acetate 0% to 20%) to give **28** as a white solid. Yield: 230 mg (99%). ^1^H-NMR (500 MHz, DMSO-*d*_6_) δ ppm 8.02 (d, *J* = 7.7 Hz, 2H, ArH-3″, ArH-5″), 7.77 (m, 2H, ArH-4″, ArH-7), 7.67 (m, 2H, phenyl-sulfonyl ArH-2″, phenyl-sulfonyl ArH-6″), 7.43–7.30 (m, 7H, ArH-4, ArH-6, benzyl ArH-2,3,4,5,6), 7.23 (t, *J* = 7.4 Hz, 1H, ArH-5), 5.09 (m, 2H, benzyl CH_2_), 3.65–3.50 (m, 4H, pyrrolidine H-2′, pyrrolidine H-5′), 2.25–2.09 (m, 2H, pyrrolidine H-4′). ^13^C-NMR (125 MHz, DMSO-*d*_6_) δ ppm 170.1 (C-2), 154.3 (C=O), 136.2 (benzyl C-1), 135.5 (C-7a), 134.8 (phenyl-sulfonyl C-1″), 133.9 (C-3a), 133.1 (phenyl-sulfonyl C-4″), 129.2 (C-3″, C-5″), 128.5 (benzyl C-3, benzyl C-5), 128.0 (C-6, benzyl C-4), 127.5 (benzyl C-2, benzyl C-6), 127.4 (C-2″, C-6″), 123.3 (C-4), 121.8 (C-5), 113.8 (C-7), 67.5 (benzyl CH_2_), 66.1 (C-3), 46.4 (C-2′), 46.1 (C-5′), 30.3 (C-4′). MS (DUIS+) *m*/*z* 463 [M + H]^+^.

##### Synthesis of 1-(Phenylsulfonyl)spiro[indoline-3,3′-pyrrolidin]-2-one (**17**)

To the solution of benzyl 2-oxo-1-(phenylsulfonyl)spiro[indoline-3,3′-pyrrolidine]-1′-carboxylate (**28**) (180 mg, 0.389 mmol) in methanol was added palladium (10%) on charcoal (90 mg, 5 *w*/*w* %) in an autoclave. The reactor was filled with 5 atm hydrogen gas and was stirred for 1 h at room temperature. The reaction mixture was then filtered on Celite and was evaporated under reduced pressure. The crude material was purified by flash chromatography (gradient: dichloromethane: methanol 0% to 5% methanol) to give **17** as an orange solid. Yield: 108 mg (85%). ^1^H-NMR (500 MHz, CDCl_3_) δ ppm 8.10 (d, *J* = 8.0 Hz, 2H, phenyl-sulfonyl ArH-3″, phenyl-sulfonyl ArH-5″), 7.91 (d, *J* = 8.4 Hz, 1H, phenyl-sulfonyl ArH-4″), 7.65 (t, *J* = 7.2 Hz, 1H, ArH-7), 7.53 (t, *J* = 7.2 Hz, 2H, phenyl-sulfonyl ArH-2″, phenyl-sulfonyl ArH-6″), 7.47 (d, *J* = 7.2 Hz, 1H, ArH-4), 7.32 (t, *J* = 8.0 Hz, 1H, ArH-6), 7.19 (t, *J* = 8.0 Hz, 1H, ArH-5), 3.08 (m, 1H, H-2′), 2.93 (m, 1H, pyrrolidine H-2′), 2.74–2.69 (m, 2H, pyrrolidine H-5′), 2.28–2.23 (m, 1H, pyrrolidine H-4′), 2.11–2.04 (m, 1H, pyrrolidine H-4′). ^13^C-NMR (125 MHz, DMSO-*d*_6_) δ ppm 175.2 (C-2), 166.8 (C-7a), 140.4 (phenyl-sulfonyl C-1″), 135.2 (C-3a), 133.0 (phenyl-sulfonyl C-4″), 129.5 (phenyl-sulfonyl C-3″, phenyl-sulfonyl C-5″), 128.4 (C-6), 127.1 (phenyl-sulfonyl C-2″, phenyl-sulfonyl C-6″), 122.5 (C-4), 121.7 (C-5), 109.6 (C-7), 55.8 (C-3), 48.3 (pyrrolidine C-2′), 47.2 (pyrrolidine C-5′), 33.0 (pyrrolidine C-4′). MS (DUIS+) *m*/*z* 329 [M + H]^+^. HRMS (ESI+): *m*/*z* [M + H]^+^ calcd. for C_17_H_17_N_2_O_3_S: 329.0960; found: 329.0949.

#### 4.1.2. Synthesis of 1′-(Phenylsulfonyl)spiro[indoline-3,3′-pyrrolidin]-2-one (**18**)

To the solution of 2-(phenylsulfonyl)-2,3,4,9-tetrahydro-1*H*-pyrido[3,4-*b*]indole (**29**) (312 mg, 1 mmol) in tetrahydrofuran: water (10:10 mL) was added 10 mL glacial acetic acid at 0 °C. *N*-bromosuccinimide (178 mg, 1 mmol, 1 equiv.) was added slowly in portions at 0 °C. The reaction mixture was stirred for 1.5 h at 0 °C. Afterwards the reaction was quenched at 0 °C by 10 mL concentrated sodium-carbonate solution and was stirred for 0.5 h, allowing to warm up to room temperature. The mixture was extracted using ethyl acetate (2 × 10 mL). The combined organic phases were washed with concentrated sodium-hydrogen carbonate (2 × 10 mL) and by brine (2 × 10 mL). The organic phases were dried over sodium-sulfate, filtered and evaporated under reduced pressure. The crude product was purified using flash chromatography (gradient: hexane: ethyl acetate 0% to 20% ethyl acetate) to afford **18** as a colorless solid. Yield: 180 mg (55%). ^1^H-NMR (500 MHz, DMSO-*d*_6_) δ ppm 10.47 (s, 1H, NH), 7.87 (d, *J =* 7.3 Hz, 2H, phenyl-sulfonyl ArH-3″, ArH-5″), 7.76 (t, *J =* 7.3 Hz, 1H, phenyl-sulfonyl ArH-4″), 7.67 (t, *J =* 7.3 Hz, 2H, phenyl-sulfonyl ArH-2″, phenyl-sulfonyl ArH-6″), 7.15 (t, *J =* 7.3 Hz, 1H, ArH-4), 6.82 (t, *J =* 8.5 Hz, 2H, ArH-5, ArH-6), 6.69 (d, *J =* 7.3 Hz, 1H, ArH-7), 3.56 (t, *J =* 6.7 Hz, 1H, pyrrolidine H-2′), 3.35 (t, *J =* 7.3 Hz, 1H, pyrrolidine H-2′), 3.35 (d, *J =* 3.5 Hz, 2H, pyrrolidine H-5′), 2.10–2.05 (m, 1H, pyrrolidine H-4′), 1.94–1.88 (m, 1H, pyrrolidine H-4′). ^13^C-NMR (125 MHz, DMSO-*d*_6_) δ ppm 181.4 (C-2), 167.0 (C-3a), 141.3 (C-7a), 135.8 (phenyl-sulfonyl C-1″), 133.3 (phenyl-sulfonyl C-4″), 129.5 (phenyl-sulfonyl C-3″, phenyl-sulfonyl C-5″), 128.3 (C-6), 127.4 (phenyl-sulfonyl C-2″, phenyl-sulfonyl C-6″), 122.4 (C-4), 121.8 (C-5), 109.6 (C-7), 55.6 (C-3), 47.4 (pyrrolidine C-2′, pyrrolidine C-5′), 35.8 (pyrrolidine C-4′). MS (DUIS+) *m*/*z* 329 [M + H]^+^. HRMS (ESI+): *m*/*z* [M + H]^+^ calcd. for C_17_H_17_N_2_O_3_S: 329.0960; found: 329.0957.

#### 4.1.3. Procedures to Afford 1′-Benzyl-1-(phenylsulfonyl)spiro[indoline-3,3′-pyrrolidine] (**39a**)

To the solution of 1′-benzylspiro[indoline-3,3′-pyrrolidin]-2-one (**37a**) (500 mg, 1.7985 mmol) in 15 mL absolutized tetrahydrofuran was added borane/tetrahydrofuran (1M) complex solution (4.495 mL, 4.495 mmol, 2.5 equiv.) and the reaction mixture was refluxed overnight. The reaction was allowed to cool down to room temperature and was quenched using 30 mL concentrated ammonium-chloride solution and was stirred for 20 min. Afterwards, 20 mL ethyl acetate was used for extraction. The combined organic phases were dried over sodium-sulfate, filtered and evaporated under reduced pressure to afford 400 mg **38a**, used as a crude product in the further steps without purification. To the solution of crude 1′-benzylspiro[indoline-3,3′-pyrrolidine] (**38a**) (400 mg 1.513 mmol) (in anhydrous dichloromethane was added triethylamine (0.419 mL, 3.03 mmol, 2 equiv.) and *N*,*N*-dimethylpyridin-4-amine (10 mg, 0.076 mmol, 0.5 equiv.) at room temperature. Afterwards benzenesulfonyl chloride (0.203 mL, 1.59 mmol, 1.05 equiv.) was added to the solution at 5 °C. The reaction mixture was stirred at room temperature overnight. The reaction was quenched using 10 mL 10% HCl solution and was extracted by 10 mL dichloromethane. The dichloromethane phase was collected and dried over sodium-sulfate. The drying agent was filtered-off and the solution was evaporated under reduced pressure. The crude product was purified by flash chromatography to afford **39a** as yellow oil. Yield: 621 mg (85% calculated to **37a**). ^1^H-NMR (500 MHz, DMSO-*d*_6_) δ ppm 7.73 (d, *J* = 7.6 Hz, 2H, phenyl-sulfonyl H-2″, phenyl-sulfonyl H-6″), 7.58 (t, *J* = 7.6 Hz, 1H, H-6), 7.46 (t, *J* = 7.9 Hz, 3H, phenyl-sulfonyl H-3″, phenyl-sulfonyl H-4″, phenyl-sulfonyl H-5″), 7.31 (t, *J* = 7.6 Hz, 2H, phenyl-sulfonyl H-3″, phenyl-sulfonyl H-5″), 7.27–7.21 (m, 5H, benzyl H-1-5), 7.05 (t, *J* = 7.6 Hz, 1H, H-5), 3.89 (d, *J* = 10.9 Hz, 1H, H-2), 3.78 (d, *J* = 10.9 Hz, 1H, H-2), 3.52 (s, 2H, benzyl CH_2_), 2.69 (m, 1H, pyrrolidine H-2′), 2.57 (m, 1H, pyrrolidine H-2′), 2.24 (d, *J* = 9.1 Hz, 1H, pyrrolidine H-5′), 2.15 (d, *J* = 9.2 Hz, 1H, pyrrolidine H-5′), 1.91–1.85 (m, 1H, pyrrolidine H-4′), 1.78–1.73 (m, 1H, pyrrolidine H-4′). ^13^C-NMR (125 MHz, DMSO-*d*_6_) δ ppm 141.0 (C-3a), 139.0 (C-7a), 134.2 (benzyl C-1), 129.8 (phenyl-sulfonyl C-1″), 129.8 (phenyl-sulfonyl C-4″), 128.7 (phenyl-sulfonyl C-3″, phenyl-sulfonyl C-5″), 128.7 (benzyl C-3, benzyl C-5), 127.4 (benzyl C-2, benzyl C-6), 127.4 (C-6), 124.9 (phenyl-sulfonyl C-2″, phenyl-sulfonyl C-6″, benzyl C-4), 124.2 (C-4), 121.8 (C-5), 114.6 (C-7), 66.7 (pyrrolidine C-2′), 63.5 (benzyl CH_2_), 59.3 (C-3), 53.3 (pyrrolidine C-5′), 49.8 (C-4′). MS (DUIS+) *m*/*z* 405 [M + H]^+^. HRMS (ESI+): *m*/*z* [M + H]^+^ calcd. for C_24_H_25_N_2_O_2_S: 405.1637; found: 405.1620.

#### 4.1.4. Procedures to Afford 1-(Phenylsulfonyl)-1′-(pyridin-4-ylmethyl)spiro[indoline-3,3′-pyrrolidine] (**39b**)

##### Synthesis of 1′-(Pyridin-4-ylmethyl)spiro[indoline-3,3′-pyrrolidin]-2-one (**37b**)

To the solution of the crude 2-(pyridin-4-ylmethyl)-2,3,4,9-tetrahydro-1*H*-pyrido[3,4-*b*]indole (**36b**) (812 mg) in tetrahydrofuran:water (10:10 mL) was added 10 mL glacial acetic acid at 0 °C. *N*-bromosuccinimide (549 mg, 3.0874 mmol) was added slowly in portions at 0 °C. The reaction mixture was stirred for 1.5 h at 0 °C. Afterwards the reaction was quenched at 0 °C by 20 mL concentrated sodium-carbonate solution and was stirred for 0.5 h, allowing to warm up to room temperature. The mixture was extracted using ethyl-acetateethyl acetate (2 × 10 mL). The combined organic phases were washed with concentrated sodium-hydrogen carbonate (2 × 10 mL) and by brine (2 × 10 mL). The organic phases were dried over sodium-sulfate, filtered and evaporated under reduced pressure. The crude product was purified using flash chromatography (gradient: dichloromethane: methanol 0% to 10% methanol) to afford **37b** as a yellow solid. Yield: 293 mg (35% calculated to **25**). ^1^H-NMR (500 MHz, DMSO-*d*_6_) δ ppm 11.63 (s, 1H, NH), 8.52 (d, *J* = 5.6 Hz, 2H, pyridine ArH-2, pyridine ArH-6), 7.59 (d, *J =* 7.2 Hz, 1H, ArH-4), 7.40 (d, *J =* 8.0 Hz, 1H, ArH-7), 7.32 (d, *J* = 5.5 Hz, 2H, pyridine ArH-3, pyridine ArH-5), 7.23 (t, *J* = 7.6 Hz, 1H, ArH-6), 7.05 (t, *J* = 7.3 Hz, 1H, ArH-5), 4.72 (s, 2H, pyridyl-methyl CH_2_), 3.65 (t, *J* = 6.7 Hz, 2H, pyrrolidine H-2′), 3.31 (m, 2H, pyrrolidine H-5′), 3.02 (t, *J* = 6.7 Hz, 2H, pyrrolidine H-4′). ^13^C-NMR (125 MHz, DMSO-*d*_6_) δ ppm 179.3 (C=O), 148.5 (pyridine C-2, pyridine C-6), 147.2 (pyridine C-1), 141.6 (C-7a), 134.7 (C-3a), 128.1 (C-6), 123.5 (C-4), 122.4 (pyridine C-3, pyridine C-5), 109.7 (C-7), 61.2 (pyridyl-methylene CH_2_), 60.1 (C-3), 54.4 (pyrrolidine C-2′), 53.6 (pyrrolidine C-5′), 34.7 (pyrrolidine C-4′). MS (DUIS+) *m*/*z* 280 [M + H]^+^.

##### Synthesis of 1-(Phenylsulfonyl)-1′-(pyridin-4-ylmethyl)spiro[indoline-3,3′-pyrrolidine] (**39b**)

To the solution of 1′-(pyridin-4-ylmethyl)spiro[indoline-3,3′-pyrrolidin]-2-one (**37b**) (289 mg, 1.05 mmol) in 10 mL anhydrousdry tetrahydrofuran was added borane/tetrahydrofuran (1 M) complex solution (2.63 mL, 2.63 mmol, 2.5 equiv.) and the reaction mixture was refluxed overnight. The reaction was allowed to cool down to room temperature and was quenched using 15 mL concentrated ammonium-chloride solution and was stirred for 20 min. Afterwards, 10 mL ethyl acetate was used for extraction. The combined organic phases were dried over sodium-sulfate, filtered and evaporated under reduced pressure to afford 292 mg **38b**, used as a crude product in the further steps without purification. To the solution of crude 1′-(pyridin-4-ylmethyl)spiro[indoline-3,3′-pyrrolidine] (**38b**) (0.292 mg) in 5 mL anhydrous dichloromethane was added triethylamine (0.307 mL, 2.22 mmol) and *N*,*N*-dimethylpyridin-4-amine (6.8 mg, 0.0555 mmol) at room temperature. Afterwards benzenesulfonyl chloride (204 mg, 1.161 mmol) was added to the solution at 5 °C. The reaction mixture was stirred at room temperature overnight. The reaction was quenched using 10 mL 10% HCl solution and was extracted by 5 mL dichloromethane. The dichloromethane phase was collected and dried over sodium-sulfate. The drying agent was filtered-off and the solution was evaporated under reduced pressure. The crude product was purified by flash chromatography to afford **39b** as yellow solid. Yield: 0.003 mg. ^1^H-NMR (500 MHz, DMSO-*d*_6_) δ ppm 8.50 (d, *J =* 6.2 Hz, 2H, pyridine ArH-2, pyridine ArH-6), 7.72 (d, *J* = 7.9 Hz, 2H, phenyl-sulfonyl ArH-2″, phenyl-sulfonyl ArH-6″), 7.58–7.53 (m, 3H, pyridine ArH-3, pyridine ArH-5, phenyl-sulfonyl ArH-4″), 7.49 (d, *J* = 7.8 Hz, 1H, ArH-7), 7.43 (t, *J* = 8.0 Hz, 2H, phenyl-sulfonyl ArH-3″, phenyl-sulfonyl ArH-5″), 7.28–7.23 (m, 2H, ArH-4, ArH-6), 7.07 (t, *J* = 7.1 Hz, 1H, ArH-5), 3.90 (d, *J =* 11.1 Hz, 1H, pyridyl methyl CH_2_), 3.83 (d, *J* = 11.1 Hz, 1H, pyridyl methyl CH_2_), 3.74 (d, *J* = 15.2 Hz, 1H, H-2), 3.67 (d, *J* = 15.2 Hz, 1H, H-2), 2.77 (m, 1H, pyrrolidine H-2′), 2.62 (m, 1H, pyrrolidine H-2′), 2.17 (m, 2H, pyrrolidine H-5′), 1.95–1.89 (m, 1H, pyrrolidine H-4′), 1.82–1.78 (m, 1H, pyrrolidine H-4′). ^13^C-NMR (125 MHz, DMSO-*d*_6_) δ ppm 148.6 (pyridine C-2, pyridine C-6), 141.0 (pyridine C-1), 143.9 (C-3a), 143.1 (C-7a), 139.5 (phenyl-sulfonyl C-1″), 134.1 (phenyl-sulfonyl C-4″), 130.0 (phenyl-sulfonyl C-3,” phenyl-sulfonyl C-5″), 128.1 (C-6), 127.3 (phenyl-sulfonyl C-2″, phenyl-sulfonyl C-6”), 127.6 (C-4), 124.2 (pyridine C-3, pyridine C-5), 124.8 (C-5), 114.1 (C-7), 65.6 (pyrrolidine C-2′), 61.5 (benzyl CH_2_), 60.1 (C-2), 53.0 (C-3), 51.3 (pyrrolidine C-5′), 32.9 (pyrrolidine C-4′). MS (DUIS+) *m*/*z* 406 [M + H]^+^. HRMS (ESI+): *m*/*z* [M + H]^+^ calcd. for C_23_H_24_N_3_O_2_S: 406.1589; found: 406.1586.

#### 4.1.5. Synthesis of 1′-Methyl-1-(phenylsulfonyl)spiro[indoline-3,3′-pyrrolidine] (**40a**)

The solution of 1′-benzyl-1-(phenylsulfonyl)spiro[indoline-3,3′-pyrrolidine] (**39a**) (2 mg, 0.495 mmol) in 5 mL methanol was filled into an autoclave. Palladium (10%) on charcoal (100 mg, 5 *w*/*w* %) was added to the solution and reaction was stirred overnight under 5 bar hydrogen atmosphere at 50 °C. The reaction mixture was filtered through a pad of Celite and was evaporated under reduced pressure. The crude residual was purified by flash chromatography (gradient: dichloromethane: methanol 0% to 10% methanol) to afford **40a** as a colorless solid. Yield: 37 mg (23%). ^1^H-NMR (500 MHz, DMSO-*d*_6_) δ ppm 7.79 (d, *J* = 7.5 Hz, 2H, phenyl-sulfonyl ArH-2″, phenyl-sulfonyl ArH-6″), 7.66 (t, *J* = 7.2 Hz, 1H, phenyl-sulfonyl ArH-4″), 7.56 (t, *J* = 7.9 Hz, 2H, phenyl-sulfonyl ArH-3″, phenyl-sulfonyl ArH-5″), 7.48 (d, *J* = 7.9 Hz, 1H, ArH-7), 7.22 (m, 2H, ArH-4, H-6), 7.03 (t, *J* = 7.9 Hz, 1H, ArH-5), 3.87 (d, *J* = 11.8 Hz, 1H, H-2), 3.75 (d, *J* = 11.8 Hz, 1H, H-2), 2.62 (m, 1H, pyrrolidine H-2′), 2.44 (m, 1H, pyrrolidine H-2′), 2.24 (d, *J* = 9.1 Hz, 1H, pyrrolidine H-5′), 2.17 (s, 3H, methyl CH_3_), 2.15 (d, *J* = 9.1 Hz, 1H, pyrrolidine H-5′), 1.85–1.80 (m, 1H, pyrrolidine H-4′), 1.74–1.69 (m, 1H, pyrrolidine H-4′). ^13^C-NMR (125 MHz, DMSO-*d*_6_) δ ppm 143.1 (C-3a, C-7a), 133.1 (phenyl-sulfonyl C-1″, phenyl-sulfonyl C-4″), 129.2 (phenyl-sulfonyl C-3″, phenyl-sulfonyl C-5″), 127.5 (C-6, phenyl-sulfonyl C-2″, phenyl-sulfonyl C-6″), 124.1 (C-4), 114.5 (C-5), 110.0 (C-7), 69.1 (pyrrolidine C-2′), 60.2 (pyrrolidine C-5′), 55.5 (C-3), 52.8 (C-2), 42.3 (pyrrolidine N(1′)-methyl), 32.5 (pyrrolidine C-4′). MS (DUIS+) *m*/*z* 329 [M + H]^+^. HRMS (ESI+): *m*/*z* [M + H]^+^ calcd. for C_18_H_21_N_2_O_2_S: 343.1324; found: 343.1320.

#### 4.1.6. Synthesis of 1′-Ethyl-1-(phenylsulfonyl)spiro[indoline-3,3′-pyrrolidine] (**40b**)

The solution of 1′-benzyl-1-(phenylsulfonyl)spiro[indoline-3,3′-pyrrolidine] (**39a**) (100 mg, 0.2475 mmol) in 5 mL methanol was filled into an autoclave. Palladium (10%) on charcoal (500 mg, 5 *w*/*w* %) was added to the solution and reaction was stirred overnight under 5 bar hydrogen atmosphere at 50 °C. The reaction mixture was filtered through a pad of Celite and was evaporated under reduced pressure. The crude residual was purified by flash chromatography (gradient: dichloromethane: methanol 0% to 10% methanol) to afford **40b** as a colorless solid. Yield: 19 mg (22%). ^1^H-NMR (500 MHz, DMSO-*d*_6_) δ ppm 7.78 (m, 2H, phenyl-sulfonyl ArH-3″, phenyl-sulfonyl ArH-5″), 7.66 (m, 1H, phenyl-sulfonyl ArH-4″), 7.56 (m, 2H, phenyl-sulfonyl ArH-2″, phenyl-sulfonyl ArH-6″), 7.50–7.47 (m, 1H, ArH-7), 7.24–7.21 (m, 2H, ArH-4, ArH-6), 7.04 (t, *J* = 7.6 Hz, 1H, ArH-5), 3.86 (d, *J* = 10.8 Hz, 1H, H-2), 3.75 (d, *J* = 10.8 Hz, 1H, H-2), 2.68 (m, 1H, pyrrolidine H-2′), 2.44 (m, 1H, pyrrolidine H-2′), 2.34 (q, *J* = 6.4 Hz, 2H, ethyl CH_2_), 2.24 (m, 1H, pyrrolidine H-5′), 2.11 (q, *J* = 8.9 Hz, 1H, pyrrolidine H-5′), 1.86–180 (m, 1H, pyrrolidine H-4′), 1.73–167 (m, 1H, pyrrolidine H-4′), 0.93 (t, *J* = 7.6 Hz, 3H, ethyl CH_3_). ^13^C-NMR (125 MHz, DMSO-*d*_6_) δ ppm 143.3 (C-3a, C-7a), 133.1 (phenyl-sulfonyl C-1″, phenyl-sulfonyl C-4″), 129.5 (phenyl-sulfonyl C-3″, phenyl-sulfonyl C-5″), 127.7 (C-6, phenyl-sulfonyl C-2″, phenyl-sulfonyl C-6″), 124.1 (C-4), 114.1 (C-5), 110.1 (C-7), 69.1 (pyrrolidine C-2′), 60.2 (pyrrolidine C-5′), 55.3 (C-3), 52.1 (C-2), 50.0 (pyrrolidine N(1′)-ethyl CH_2_), 32.5 (pyrrolidine C-4′), 13.6 (pyrrolidine N(1′)-ethyl CH_3_). MS (DUIS+) *m*/*z* 343 [M + H]^+^. HRMS (ESI+): *m*/*z* [M + H]^+^ calcd. for C_19_H_23_N_2_O_2_S: 343.1480; found: 343.1496.

#### 4.1.7. Procedures to Afford 1′-(2-Fluorobenzyl)-1-(phenylsulfonyl)spiro[indoline-3,3′-pyrrolidine] (**44a**)

##### Synthesis of 1′-(2-Fluorobenzyl)spiro[indoline-3,3′-pyrrolidin]-2-one (**42a**)

To the solution of **41a** (1.54 g, 5.5 mmol) in tetrahydrofuran: water (30:30 mL) was added 30 mL glacial acetic acid at 0 °C. *N*-bromosuccinimide (979 mg, 5.5 mmol, 1 equiv.) was added slowly in portions at 0 °C. The reaction mixture was stirred for 1.5 h at 0 °C. Afterwards the reaction was quenched at 0 °C by 40 mL concentrated sodium-carbonate solution and was stirred for 0.5 h, allowing to warm up to room temperature. The mixture was extracted using ethyl acetate (2 × 20 mL). The combined organic phases were washed with concentrated sodium-hydrogen carbonate (2 × 20 mL) and by brine (2 × 20 mL). The organic phases were dried over sodium-sulfate, filtered and evaporated under reduced pressure. The product **42a** was purified by flash chromatography (gradient: dichloromethane: methanol 0% to 10% methanol) Yield: 970 mg (56%). ^1^H-NMR (500 MHz, DMSO-*d*_6_) δ ppm 10.31 (s, 1H, NH), 8.01 (d, *J* = 8.8 Hz, 1H, benzyl ArH-6), 7.77 (d, *J* = 7.9 Hz, 1H, ArH-4), 7.65 (t, *J* = 7.9 Hz, 1H, benzyl ArH-4), 7.47 (t, *J* = 6.2 Hz, 1H, benzyl ArH-5), 7.24 (t, *J* = 8.4 Hz, 1H, ArH-6), 7.16 (m, 1H, benzyl ArH-3), 6.94 (t, *J* = 7.5 Hz, 1H, ArH-5), 6.80 (d, *J* = 7.5 Hz, 1H, ArH-7), 3.75 (s, 2H, benzyl CH_2_), 3.06 (m, 1H, pyrrolidine H-2′), 2.76 (d, *J =* 8.9 Hz, 1H, pyrrolidine H-5′), 2.69 (d, *J =* 8.9 Hz, 1H, pyrrolidine H-5′), 2.61 (q, *J =* 7.9 Hz, 1H, pyrrolidine H-2’), 2.20–2.15 (m, 1H, pyrrolidine H-4′), 1.92–1.87 (m, 1H, pyrrolidine H-4′). ^13^C-NMR (125 MHz, DMSO-*d*_6_) δ ppm 181.5 (C=O), 178.0 (benzyl C-2), 141.6 (C-7a), 137.0 (C-3a), 135.5 (benzyl C-6), 130.2 (benzyl C-4), 127.6 (C-6), 124.7 (benzyl C-1), 123.9 (benzyl C-5), 123.3 (C-4), 122.3 (C-5), 115.7 (benzyl C-3), 109.6 (C-7), 64.0 (C-3), 53.86 (benzyl CH_2_), 52.9 (pyrrolidine C-2′), 51.7 (pyrrolidine C-5′), 37.1 (pyrrolidine C-4′). MS (DUIS+) *m*/*z* 297 [M + H]^+^.

##### Synthesis of 1′-(2-Fluorobenzyl)-1-(phenylsulfonyl)spiro[indoline-3,3′-pyrrolidine] (**44a**)

Compound **43a** was prepared according to the same procedure as **38a**, starting from **42a** (100 mg, 0.3378 mmol), using borane-tetrahydrofuran (0.6757 mL, 0.6757 mmol, 2.5 equiv.), to afford 10 mg crude product used in the next step without further purification. Compound **44a** was prepared according to the same procedure as **39a**, starting from crude **43a** (10 mg, 0.03545 mmol), using benzylsulfonyl chloride (50 mg, 0.07445 mmol, 1.05 equiv.), triethylamine (0.010 mL, 0.0709 mmol, 2 equiv.) and *N*,*N*-dimethylpyridin-4-amine (0.2 mg, 0.0017 mmol, 0.5 equiv.). The product was purified by preparative-HPLC (gradient: water: acetonitrile 0% to 100% acetonitrile) Yield: 2 mg. ^1^H-NMR (500 MHz, CDCl_3_) δ ppm 8.22 (t, *J =* 6.7 Hz, 2H, phenyl-sulfonyl ArH-3″, phenyl-sulfonyl ArH-5″), 7.88 (m, 1H, phenyl-sulfonyl ArH-4″), 7.69 (m, 2H, phenyl-sulfonyl ArH-2″, phenyl-sulfonyl ArH-6″), 7.53 (t, *J* = 6.2 Hz, 1H, benzyl ArH-4), 7.47 (m, 1H, ArH-7), 7.33 (t, *J* = 7.8 Hz, 1H, benzyl ArH-5), 7.23 (t, *J* = 7.8 Hz, 1H, benzyl ArH-6), 7.17–7.08 (m, 2H, ArH-5, ArH-6), 6.73 (d, *J* = 7.0 Hz, 2H, benzyl ArH-3), 4.22 (m, 2H, H-2), 3.98 (m, 2H, benzyl CH_2_), 3.75 (m, 1H, pyrrolidine H-2′), 3.40 (m, 1H, pyrrolidine H-2′), 3.14 (m, 1H, pyrrolidine H-5′), 2.98 (m, 1H, pyrrolidine H-5′), 2.28 (m, 1H, pyrrolidine H-4′), 1.51 (m, 1H, pyrrolidine H-4′). ^13^C-NMR (125 MHz, CDCl_3_) δ ppm 159.3 (benzyl C-2), 144.1 (C-3a), 142.5 (C-7a), 137.3 (phenyl-sulfonyl C-1″), 134.2 (phenyl-sulfonyl C-4″), 130.3 (benzyl C-6), 129.5 (phenyl-sulfonyl C-3″, phenyl-sulfonyl C-5″), 129.2 (benzyl C-4), 128.2 (C-6), 127.6 (phenyl-sulfonyl C-2″, phenyl-sulfonyl C-6″), 125.8 (benzyl C-1), 125.3 (benzyl C-5), 125.0 (C-4), 121.6 (C-5), 115.7 (benzyl C-3), 113.0 (C-7), 62.3 (C-2′), 59.6 (benzyl CH_2_), 55.8 (C-3), 54.9 (C-5′), 53.6 (C-2), 33.1 (C-4′). MS (DUIS+) *m*/*z* 423 [M + H]^+^. HRMS (ESI+): *m*/*z* [M + H]^+^ calcd. for C_24_H_24_N_2_O_2_FS: 423.1543; found: 423.1531.

#### 4.1.8. Procedures to Afford 1′-(3-Fluorobenzyl)-1-(phenylsulfonyl)spiro[indoline-3,3′-pyrrolidine] (**44b**)

##### Synthesis of 1′-(3-Fluorobenzyl)spiro[indoline-3,3′-pyrrolidin]-2-one (**42b**)

To the solution of **41b** (1.681 g, 6 mmol) in tetrahydrofuran: water (30:30 mL) was added 30 mL glacial acetic acid at 0 °C. *N*-bromosuccinimide (1.069 g, 6 mmol, 1 equiv.) was added slowly in portions at 0 °C. The reaction mixture was stirred for 1.5 h at 0 °C. Afterwards the reaction was quenched at 0 °C by 40 mL concentrated sodium-carbonate solution and was stirred for 0.5 h, allowing to warm up to room temperature. The mixture was extracted using ethyl acetate (2 × 20 mL). The combined organic phases were washed with concentrated sodium-hydrogen carbonate (2 × 20 mL) and by brine (2 × 20 mL). The organic phases were dried over sodium-sulfate, filtered and evaporated under reduced pressure. The product **42b** was purified by flash chromatography (gradient: dichloromethane: methanol 0% to 10% methanol) Yield: 1.31 g (76% calculated to **29**). ^1^H-NMR (500 MHz, DMSO-*d*_6_) δ ppm 10.31 (s, 1H, NH), 7.38–7.33 (m, 2H, benzyl H-5, benzyl ArH-6), 7.21–7.16 (m, 3H, ArH-4, benzyl H-2, benzyl ArH-4), 7.04 (m, 1H, ArH-6), 6.98 (t, *J* = 7.0 Hz, 1H, ArH-5), 6.81 (d, *J* = 8.0 Hz, 1H, ArH-7), 3.72 (s, 2H, benzyl CH_2_), 3.07 (m, 1H, pyrrolidine H-2′), 2.74 (d, *J* = 8.8 Hz, 1H, pyrrolidine H-5′), 2.66 (d, *J* = 8.8 Hz, 1H, pyrrolidine H-2′), 2.56 (m, 1H, pyrrolidine H-5′), 2.20 (m, 1H, pyrrolidine H-4′), 1.90 (m, 1H, pyrrolidine H-4′). ^13^C-NMR (125 MHz, CDCl_3_) δ ppm 181.96 (C=O), 161.2 (benzyl C-3), 142.3 (C-7a), 141.4 (benzyl C-1), 130.4 (benzyl C-5), 127.8 (benzyl-C6, C-6), 122.1 (benzyl C-2, benzyl C-4), 109.4 (C-7), 64.7 (benzyl CH_2_), 63.9 (C-3), 53.9 (pyrrolidine C-2′), 52.7 (C-5′), 36.8 (pyrrolidine C-4′). MS (DUIS+) *m*/*z* 297 [M + H]^+^.

##### Synthesis of 1′-(3-Fluorobenzyl)-1-(phenylsulfonyl)spiro[indoline-3,3′-pyrrolidine] (**44b**)

Compound **43b** was prepared according to the same procedure as **38a**, starting from **42b** (385 mg, 1.3 mmol), using borane-tetrahydrofuran (3.250 mL, 3.25 mmolm 2.5 equiv.), to afford 338 mg crude product used in the next time without further purification. Compound **44b** was prepared according to the same procedure as **39a**, starting from crude **43b** (338 mg, 1.197 mmol), using benzylsulfonyl chloride (221 mg, 1.2579 mmol, 1.05 equiv.), triethylamine (0.331 mL, 2.396 mmol, 2 equiv.) and *N*,*N*-dimethylpyridin-4-amine (7 mg, 0.0599 mmol, 0.5 equiv.). The product was purified by preparative HPLC (gradient: dichloromethane: methanol 0% to 10% methanol) Yield: 11 mg. ^1^H-NMR (500 MHz, CDCl_3_) δ ppm 7.83 (d, *J* = 8.2 Hz, 1H, ArH-7), 7.77 (d, *J* = 7.6 Hz, 1H, benzyl ArH-6), 7.73 (d, *J* = 7.6 Hz, 1H, ArH-4), 7.64 (d, *J* = 8.2 Hz, 1H, benzyl ArH-4), 7.58–7.53 (m, 1H, benzyl ArH-5), 7.50–7.39 (m, 3H, phenyl-sulfonyl ArH-3″, phenyl-sulfonyl ArH-4″, phenyl-sulfonyl ArH-5″), 7.37–7.27 (m, 2H, phenyl-sulfonyl ArH-2″, phenyl-sulfonyl ArH-6″), 7.16–7.12 (m, 1H, phenyl-sulfonyl ArH-6), 7.09–7.02 (m, 1H, phenyl-sulfonyl ArH-5), 6.99 (m, 1H, benzyl H-2), 4.23 (m, 1H, H-2), 4.11 (m, 1H, H-2), 3.93–3.71 (m, 2H, benzyl CH_2_), 3.23 (m, 1H, pyrrolidine H-2′), 2.94 (m, 1H, pyrrolidine H-2′), 2.83 (m, 1H, pyrrolidine H-5′), 2.69 (m, 1H, pyrrolidine H-5′), 2.39–2.25 (m, 1H, pyrrolidine H-4′), 2.18 (m, 1H, pyrrolidine H-5′). ^13^C-NMR (125 MHz, CDCl_3_) δ ppm 163.0 (benzyl C-3), 143.6 (C-3a), 143.5 (C-7a), 137.6 (phenyl-sulfonyl C-1″), 134.2 (phenyl-sulfonyl C-4″), 130.4 (benzyl C-5), 129.5 (phenyl-sulfonyl C-3″, phenyl-sulfonyl C-5″), 129.1 (C-6), 128.0 (phenyl-sulfonyl C-2″, phenyl-sulfonyl C-6″), 127.7 (benzyl C-6), 125.6 (benzyl C-1), 125.3 (C-4), 124.1 (C-5), 121.8, 115.8 (benzyl C-2), 113.4 (C-7), 113.3 (benzyl C-4), 61.2 (benzyl CH_2_), 59.9 (pyrrolidine C-2′), 55.3 (C-3), 54.8 (pyrrolidine C-5′), 53.6 (C-2), 33.1 (pyrrolidine C-4’). MS (DUIS+) *m*/*z* 423 [M + H]^+^. HRMS (ESI+): *m*/*z* [M + H]^+^ calcd. for C_24_H_24_N_2_O_2_SF: 423.1543; found: 423.1558.

#### 4.1.9. Procedures to Afford 1′-(4-Fluorobenzyl)-1-(phenylsulfonyl)spiro[indoline-3,3′-pyrrolidine] (**44c**)

##### Synthesis of 1′-(4-Fluorobenzyl)spiro[indoline-3,3′-pyrrolidin]-2-one (**42c**)

To the solution of **41c** (2.361 g, 8.4321 mmol) in tetrahydrofuran: water (30:30 mL) was added 30 mL glacial acetic acid at 0 °C. *N*-bromosuccinimide (1.501 g, 8.4321 mmol, 1 equiv.) was added slowly in portions at 0 °C. The reaction mixture was stirred for 1.5 h at 0 °C. Afterwards the reaction was quenched at 0 °C by 40 mL concentrated sodium-carbonate solution and was stirred for 0.5 h, allowing to warm up to room temperature. The mixture was extracted using ethyl acetate (2 × 20 mL). The combined organic phases were washed with concentrated sodium-hydrogen carbonate (2 × 20 mL) and by brine (2 × 20 mL). The organic phases were dried over sodium-sulfate, filtered and evaporated under reduced pressure. The product **42c** was purified by flash chromatography (gradient: dichloromethane: methanol 0% to 10% methanol) Yield: 320 mg (19% calculated to **25**). ^1^H-NMR (500 MHz, DMSO-*d*_6_) δ ppm 10.30 (s, 1H, NH), 7.41–7.35 (m, 3H, benzyl H-2, benzyl ArH-6, ArH-4), 7.16 (m, 3H, benzyl ArH-3, benzyl ArH-5, ArH-6), 6.96 (t, *J* = 7.2 Hz, 1H, ArH-5), 6.81 (d, *J* = 7.4 Hz, 1H, ArH-7), 5.73 (s, 2H, benzyl CH_2_), 3.03 (m, 1H, pyrrolidine H-5′), 2.73 (d, *J* = 9.3 Hz, 1H, pyrrolidine H-2′), 2.64 (d, *J* = 9.3 Hz, 1H, pyrrolidine H-2′), 2.58–2.53 (m, 1H, pyrrolidine H-5′), 2.21–2.16 (m, 1H, pyrrolidine H-4′), 1.95–1.87 (m, 1H, pyrrolidine H-4′) ^13^C-NMR (125 MHz, DMSO-*d*_6_) δ ppm 179.1 (C=O), 161.9 (benzyl C-4), 141.6 (C-7a), 138.2 (benzyl C-1), 134.6 (C-3a), 130.7 (benzyl C-2, benzyl C-6), 128.0 (C-6), 123.3 (C-4), 122.3 (C-2), 115.4 (benzyl C-3, benzyl C-5), 109.6 (C-7), 64.0 (benzyl CH_2_), 58.3 (C-3), 54.0 (pyrrolidine C-2′), 52.9 (pyrrolidine C-5′), 37.0 (pyrrolidine C-4′). MS (DUIS+) *m*/*z* 297 [M + H]^+^.

##### Synthesis of 1′-(4-Fluorobenzyl)-1-(phenylsulfonyl)spiro[indoline-3,3′-pyrrolidine] (**44c**)

Compound **43c** was prepared according to the same procedure as **38a**, starting from **42c** (310 mg, 1.0473 mmol), using borane-tetrahydrofuran (2.620 mL, 2.620 mmol, 2.5 equiv.), to afford 461 mg crude product used in the next step without further purification. Compound **44c** was prepared according to the same procedure as **39a**, starting from crude **43c** (100 mg, 0.354 mmol), using benzylsulfonyl chloride (131 mg, 0.745 mmol, 1.05 equiv.), triethylamine (0.099 mL, 0.7092 mmol) and *N*,*N*-dimethylpyridin-4-amine (2 mg, 0.01773 mmol). The product **44c** was purified by flash chromatography (gradient: dichloromethane: methanol 0% to 10% methanol) Yield: 10 mg. ^1^H-NMR (500 MHz, CDCl3) δ ppm 7.84–7.77 (m, 2H, phenyl-sulfonyl ArH-3″, phenyl-sulfonyl ArH-5″), 7.74–7.70 (m, 1H, phenyl-sulfonyl ArH-4″), 7.67–7.57 (m, 3H, phenyl-sulfonyl ArH-2″, phenyl-sulfonyl ArH-6″, ArH-7), 7.50–7.45 (m, 2H, benzyl ArH-2, benzyl ArH-6), 7.43–7.40 (m, 1H, ArH-4), 7.24–7.20 (m, 1H, ArH-6), 7.16–7.12 (m, 2H, benzyl ArH-3, benzyl ArH-5), 7.09 (m, 1H, ArH-5), 4.39 (m, 1H, H-2), 4.29 (m, 1H, H-2), 4.18 (m, 1H, benzyl CH_2_), 413.06 (m, 1H, benzyl CH_2_), 3.90 (m, 1H, pyrrolidine H-2′), 3.70 (m, 1H, pyrrolidine H-2′), 3.06 (m, 1H), 2.68 (m, 1H, pyrrolidine H5′), 2.33 (m, 1H, pyrrolidine H-5′), 2.33 (m, 1H, pyrrolidine H-4′), 1.75 (m, 1H, pyrrolidine H-4′). ^13^C-NMR (125 MHz, CDCl_3_) δ ppm 159.8 (benzyl C-4), 144.1 (C-3a), 142.6 (C-7a), 137.5 (benzyl C-1), 134.2 (phenyl-sulfonyl C-1″, phenyl-sulfonyl C-4″), 130.7 (benzyl C-2, benzyl C-6), 129.2 (phenyl-sulfonyl C-3″, phenyl-sulfonyl C-5″), 128.3 (C-6), 127.7 (phenyl-sulfonyl C-2″, phenyl-sulfonyl C-6″), 125.8 (C-4), 125.2 (C-5), 115.5 (benzyl C-3, benzyl C-5), 113.1 (C-7), 62.4 (pyrrolidine C-2′), 59.8 (benzyl CH_2_), 55.5 (C-3), 55.0 (pyrrolidine C-5′), 53.7 (C-2), 33.0 (pyrrolidine C-4′). MS (DUIS+) *m*/*z* 423 [M + H]^+^. HRMS (ESI+): *m*/*z* [M + H]^+^ calcd. for C_24_H_24_N_2_O_2_SF: 423.1543; found: 423.1542.

#### 4.1.10. Synthesis of 1′-Benzyl-1-((2-fluorophenyl)sulfonyl)spiro[indoline-3,3′-pyrrolidine] (**46a**)

Compound **46a** was prepared according to the same procedure as **39a**, starting from crude **38a** (15 mg, 0.0567 mmol), using 2-fluorobenzylsulfonyl chloride (23 mg, 0.11819 mmol), triethylamine (0.015 mL, 0.1188 mmol, 2.1 equiv.) and *N*,*N*-dimethylpyridin-4-amine (0.7 mg, 0.006 mmol, 0.1 equiv.). The product was purified by flash chromatography (gradient: dichloromethane: methanol 0% to 10% methanol) Yield: 6.5 mg. ^1^H-NMR (500 MHz, CDCl_3_) δ ppm 8.00 (t, *J* = 6.7 Hz, 1H, phenyl-sulfonyl ArH-4″), 7.53 (m, 3H, phenyl-sulfonyl ArH-3″, phenyl-sulfonyl ArH-6″, ArH-7), 7.41–7.34 (m, 5H, benzyl ArH-2, benzyl ArH-3, benzyl ArH-5, benzyl ArH-6, ArH-4), 7.27 (t, *J =* 8.1 Hz, 1H, phenyl-sulfonyl ArH-5″), 7.17 (t, *J* = 8.1 Hz, 1H, benzyl ArH-4), 7.10 (t, *J* = 9.4 Hz, 1H, ArH-6), 7.04 (t, *J* = 7.6 Hz, 1H, ArH-5), 4.16–4.07 (m, 5H, benzyl CH_2_, H-2, pyrrolidine H-2′), 3.34 (m, 1H, pyrrolidine H-2′), 3.17 (m, 1H, pyrrolidine H-5′), 3.05 (m, 1H, pyrrolidine H-5′), 2.36 (m, 1H, pyrrolidine H-4′), 2.14 (m, 1H, pyrrolidine H-4′). ^13^C-NMR (125 MHz, DMSO-*d*_6_) δ ppm 155.6 (phenyl-sulfonyl C-2″), 149.7 (C-3a), 142.8 (C-7a), 133.2 (benzyl C-1), 131.8 (phenyl-sulfonyl C-4″), 131.0 (phenyl-sulfonyl C-6″), 129.6 (benzyl C-3, benzyl C-5), 128.4 (benzyl C-2, benzyl C-6), 124.2 (phenyl-sulfonyl C-5″), 122.9 (benzyl C-4), 122.5 (C-4), 121.0 (C-5), 120.4 (phenyl-sulfonyl C-1″), 116.8 (phenyl-sulfonyl C-3″), 111.3 (C-7), 63.2 (pyrrolidine C-2′), 62.6 (benzyl CH_2_), 55.1 (C-3), 52.5 (pyrrolidine C-5′), 49.2 (C-2), 37.1 (pyrrolidine C-4′). MS (DUIS+) *m*/*z* 423 [M + H]^+^. HRMS (ESI+): *m*/*z* [M + H]^+^ calcd. for C_24_H_24_N_2_O_2_SF: 423.1543; found: 423.1550.

#### 4.1.11. Synthesis of 1′-Benzyl-1-((3-fluorophenyl)sulfonyl)spiro[indoline-3,3′-pyrrolidine] (**46b**)

Compound **45b** was prepared according to the same procedure as **39a**, starting from crude **38a** (15 mg, 0.0567 mmol), using 3-fluorobenzylsulfonyl chloride (23 mg, 0.1188 mmol, 2.1 equiv.), triethylamine (0.015 mL, 0.1188 mmol, 2.1 equiv.) and *N*,*N*-dimethylpyridin-4-amine (0.7 mg, 0.006 mmol, 0.1 equiv.). The product was purified by flash chromatography (gradient: dichloromethane: methanol 0% to 10% methanol) Yield: 5.5 mg. ^1^H-NMR (500 MHz, CDCl_3_) δ ppm 8.25 (m, 1H, phenyl-sulfonyl ArH-2″), 7.65–7.58 (m, 2H, phenyl-sulfonyl ArH-5″, phenyl-sulfonyl ArH-6″), 7.48–7.38 (m, 6H, ArH-7, benzyl ArH-2–6), 7.29–7.25 (m, 2H, ArH-4, ArH-6), 7.21 (m, 1H, phenyl-sulfonyl ArH-4″), 7.08 (t, *J =* 7.6 Hz, 1H, ArH-5), 4.16 (d, *J =* 12.7 Hz, 1H, benzyl CH_2_), 4.09 (d, *J =* 12.7 Hz, 1H, benzyl CH_2_), 3.99 (d, *J =* 10.9 Hz, 1H, H-2), 3.85 (d, *J =* 10.9 Hz, 1H, H-2), 3.33–3.24 (m, 2H, pyrrolidine H-2′), 3.12 (d, *J =* 11.6 Hz, 1H, pyrrolidine H-5′), 3.00 (d, *J =* 11.6 Hz, 1H, pyrrolidine H-5′), 2.26–2.20 (m, 1H, pyrrolidine H-4′), 2.05–2.00 (m, 1H, pyrrolidine H-4′). ^13^C-NMR (125 MHz, CDCl_3_) δ ppm 158.6 (phenyl-sulfonyl C-3″), 146.8 (C-3a), 145.4 (C-7a), 137.5 (benzyl C-1), 130.5 (phenyl-sulfonyl C-1″), 129.7 (phenyl-sulfonyl C-5″), 129.0 (benzyl C-3, benzyl C-5), 128.7 (benzyl C-2, benzyl C-6), 124.5 (C-6), 122.8 (benzyl C-4), 122.7 (phenyl-sulfonyl C-6″), 121.7 (C-4), 120.2 (C-5), 117.7 (phenyl-sulfonyl C-4″), 114.3 (phenyl-sulfonyl C-2″), 109.5 (C-7), 63.0 (pyrrolidine C-2′), 62.6 (benzyl CH_2_), 55.4 (C-3), 52.7 (pyrrolidine C-5′), 49.2 (C-2), 37.9 (pyrrolidine C-4′). MS (DUIS+) *m*/*z* 423 [M + H]^+^. HRMS (ESI+): *m*/*z* [M + H]^+^ calcd. for C_24_H_24_N_2_O_2_SF: 423.1543; found: 423.1547.

#### 4.1.12. Synthesis of 1′-Benzyl-1-((4-fluorophenyl)sulfonyl)spiro[indoline-3,3′-pyrrolidine] (**46c**)

Compound **46c** was prepared according to the same procedure as **39a**, starting from crude **38a** (15 mg, 0.0567 mmol), using 4-fluorobenzylsulfonyl chloride (23 mg, 0.1182 mmol, 2.1 equiv.), triethylamine (0.015 mL, 0.1188 mmol, 2.1 equiv.) and *N*,*N*-dimethylpyridin-4-amine (0.7 mg, 0.006 mmol, 0.1 equiv.). The product was purified by flash chromatography (gradient: dichloromethane: methanol 0% to 10% methanol) Yield: 7.7 mg. ^1^H-NMR (500 MHz, CDCl_3_) δ ppm 7.80 (m, 2H, phenyl-sulfonyl ArH-3″, phenyl-sulfonyl ArH-5″), 7.63 (d, *J* = 7.9 Hz, 1H, ArH-7), 7.47 (m, 2H, benzyl H-3, benzyl ArH-5), 7.40 (m, 3H, benzyl ArH-2, benzyl ArH-4, benzyl ArH-6), 7.28 (m, 2H, phenyl-sulfonyl ArH-3″, phenyl-sulfonyl ArH-5″), 7.09–7.05 (m, 3H, ArH-4, ArH-5, ArH-6), 4.10 (d, *J* = 12.3 Hz, 1H, H-2), 4.02–3.96 (m, 2H, benzyl CH_2_), 3.84 (d, *J* = 11.3 Hz, 1H, H-2), 3.25 (m, 1H, pyrrolidine H-2′), 3.16 (m, 1H, pyrrolidine H-2′), 2.94 (m, 1H, pyrrolidine H-5′), 2.89 (m, 1H, pyrrolidine H-5′), 2.24–2.18 (m, 1H, pyrrolidine H-4′), 2.03–1.99 (m, 1H, pyrrolidine H-4′). ^13^C-NMR (125 MHz, CDCl_3_) δ ppm 163.7 (phenyl-sulfonyl C-4″), 152.7 (C-3a), 150.6 (C-7a), 147.0 (benzyl C-1), 129.6 (phenyl-sulfonyl C-1″), 129.5 (phenyl-sulfonyl C-2″, phenyl-sulfonyl C-6″), 129.2 (benzyl C-3, benzyl C-5), 129.1 (benzyl C-2, benzyl C-6), 128.8 (C-6), 128.6 (benzyl C-4), 125.6 (C-4), 124.4 (C-5), 122.8 (phenyl-sulfonyl C-3″, phenyl-sulfonyl C-5″), 116.1 (C-7), 63.1 (pyrrolidine C-2′), 62.4 (benzyl CH_2_), 54.1 (C-3), 52.8 (pyrrolidine C-5′), 49.1 (pyrrolidine C-2′), 36.6 (pyrrolidine C-4′). MS (DUIS+) *m*/*z* 423 [M + H]^+^. HRMS (ESI+): *m*/*z* [M + H]^+^ calcd. for C_24_H_24_N_2_O_2_SF: 423.1543; found: 423.1563.

### 4.2. General Procedure for the Serotonergic Screening Assays

#### 4.2.1. Cell Culture and Preparation of Cell Membranes

HEK293 cells with stable expression of human serotonin 5-HT_1A_, 5-HT_6_ or 5-HT_7b_ receptors (all prepared with the use of Lipofectamine 2000) or CHO-K1 cells with plasmid containing the sequence coding for the human serotonin 5-HT_2A_ receptor (PerkinElmer) were maintained at 37 °C in a humidified atmosphere with 5% CO_2_ and were grown in Dulbeco’s Modifier Eagle Medium containing 10% dialysed fetal bovine serum and 500 μg/mL G418 sulphate. For membranes preparations, cells were subcultured in 150 cm^2^ flasks, grown to 90% confluence, washed twice with prewarmed to 37 °C phosphate buffered saline (PBS) and were pelleted by centrifugation (200 g) in PBS containing 0.1 mM EDTA and 1 mM dithiothreitol. Prior to membrane preparations pellets were stored at −80 °C.

#### 4.2.2. Radioligand Binding Assays

Cell pellets were thawed and homogenized in 10 volumes of assay buffer using an Ultra Turrax tissue homogenizer and centrifuged twice at 35,000 g for 20 min at 4 °C, with incubation for 15 min at 37 °C in between. The composition of the assay buffers was as follows: for 5-HT_1A_R: 50 mM Tris–HCl, 0.1 mM EDTA, 4 mM MgCl_2_, 10 µM pargyline and 0.1% ascorbate; for 5-HT_2A_R: 50 mM Tris–HCl, 0.1 mM EDTA, 4 mM MgCl_2_ and 0.1% ascorbate; for 5-HT_6_R: 50 mM Tris–HCl, 0.5 mM EDTA and 4 mM MgCl_2_, for 5-HT_7b_R: 50 mM Tris–HCl, 4 mM MgCl_2_, 10 µM pargyline and 0.1% ascorbate. All assays were incubated in a total volume of 200 μL in 96-well microtiter plates for 1 h at 37 °C, except for 5-HT_1A_R and 5-HT_2A_R which were incubated at room temperature for 1 h and 1.5 h respectively. The process of equilibration is terminated by rapid filtration through Unifilter plates with a 96-well cell harvester and radioactivity retained on the filters was quantified on a Microbeta plate reader (PerkinElmer).

For displacement studies the assay samples contained as radioligands: 2.5 nM [^3^H]-8-OH-DPAT (5002.4 GBq/mmol) for 5-HT_1A_R; 1 nM [^3^H]-Ketanserin (1975.8 GBq/mmol) for 5-HT_2A_R; 2 nM [^3^H]-LSD (3093.2 GBq/mmol) for 5-HT_6_R or 0.8 nM [^3^H]-5-CT (1450.4 GBq/mmol) for 5-HT_7_R. Non-specific binding is defined with 10 µM of 5-HT in 5-HT_1A_R and 5-HT_7_R binding experiments, whereas 10 µM of chlorpromazine or 10 µM of methiothepine were used in 5-HT_2A_R and 5-HT_6_R assays, respectively. Each compound was tested in triplicate at 7–8 concentrations (10^−11^–10^−4^ M). The inhibition constants (*K*_i_) were calculated from the Cheng-Prusoff equation [47]. Results were expressed as means of at least three separate experiments (SD ≤ 24%).

#### 4.2.3. Docking Procedure

The homology model of h-5HT_6_R [15] was prepared for docking experiment my Schrödinger’s Protein Preparation Wizard by default settings for protomer-state optimization and restrained minimization by OPLS_2005 force field. Single precision docking was performed with Schrödinger’s Glide by default settings. The ligand (**46a**) was prepared for docking by Schrödinger’s LigPrep, generating possible ionization states (at pH range of 7.0 ± 2.0), tautomers and stereoisomers. Constrained docking was set to two required interactions (at least 1 match): hydrogen bond formed with Asn288^6.55^ and/or hydrogen bond formed with Ser193^5.43^.

## Figures and Tables

**Table 1:** Serotonergic G-protein coupled receptor (GPCR) panel of **6** as measured in binding assays of four serotonin receptors (*K*_i_ values are in µM).

ID	Structure	5-HT_1A_	5-HT_2A_	5-HT_6_	5-HT_7_
**6**	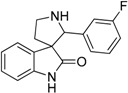	16.91	5.05	6.75	8.48

**Table 2:** Serotonergic GPCR panel of derivatives substituted in the 2′-phenyl moiety as measured in binding assays of four serotonin receptors (*K*_i_ values are in µM).

ID	Structure	5-HT_1A_	5-HT_2A_	5-HT_6_	5-HT_7_
**7**	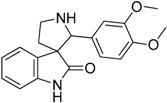	7.07	5.45	**2.26**	2.69
**8**	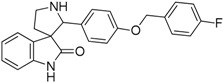	1.74	2.28	**3.18**	1.09
**9**	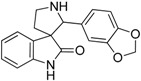	9.84	2.69	**3.20**	8.82
**10**	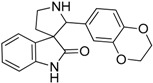	12.08	4.33	**4.11**	12.74
**11**	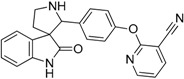	49.11	8.68	**6.20**	58.96
**12**	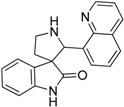	45.67	1.12	**6.63**	10.45
**13**	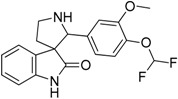	7.14	5.77	**7.73**	7.49
**14**	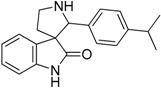	7.76	4.54	**8.62**	8.98
**15**	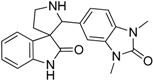	40.50	23.70	**9.49**	46.72
**16**	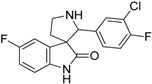	not active	53.89	**16.54**	not active

**Table 3:** Serotonergic GPCR panel of the *N*-phenylsulfonylated derivatives at N′ and N-positions as measured in binding assays of four serotonin receptors (*K*_i_ values are in µM).

ID	Structure	5-HT_1A_	5-HT_2A_	5-HT_6_	5-HT_7_
**17**	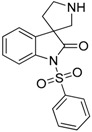	not active	not active	**12.30**	58.31
**18**	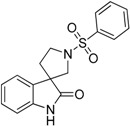	not measured	87.35	**6.86**	not active

**Table 4:** Serotonergic GPCR panel of *N*-phenylsulfonyl indolines with different N′-substituents as measured in binding assays of four serotonin receptors (*K*_i_ values are in µM).

ID	Structure	5-HT_1A_	5-HT_2A_	5-HT_6_	5-HT_7_
**39a**	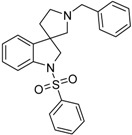	not measured	4.76	**0.76**	55.76
**39b**	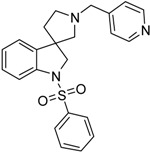	not active	26.85	**6.60**	not active
**40a**	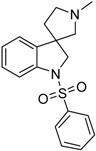	not measured	4.52	**1.85**	40.17
**40b**	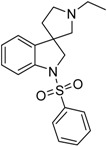	not measured	3.82	**2.42**	not active

**Table 5:** Serotonergic GPCR panel of the fluorobenzyl derivatives as measured in binding assays of three serotonin receptors (*K*_i_ values are in µM).

ID	Structure	5-HT_1A_	5-HT_6_	5-HT_7_
**44a**	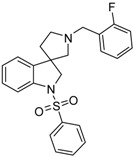	not active	**4.53**	78.93
**44b**	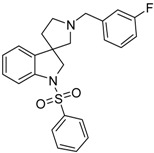	not active	**1.97**	31.49
**44c**	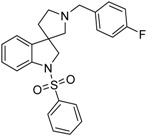	not active	**1.77**	15.74

**Table 6:** Serotonergic GPCR panel of the fluorophenylsulfonyl derivatives as measured in binding assays of three serotonin receptors (*K*_i_ values are in µM).

ID	Structure	5-HT_1A_	5-HT_6_	5-HT_7_
**46a**	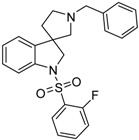	not active	**0.19**	42.57
**46b**	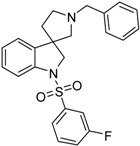	not active	**0.71**	76.00
**46c**	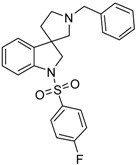	not active	**2.45**	56.05
